# Is White Evangelical Antistructural Theology Related to Poor Health Outcomes?

**DOI:** 10.1111/1468-0009.12688

**Published:** 2024-01-19

**Authors:** DAVID A. KINDIG, YASMIN MOHD ARIFFIN, HANNAH OLSON‐WILLIAMS

**Affiliations:** ^1^ Population Health Institute, University of Wisconsin School of Medicine and Public Health

**Keywords:** religion, evangelical, population health outcomes, outcomes, cultural factors, structural factors

## Abstract

**Policy Points**

- White evangelical theology has an “antistructural” component.
- Counties with a high percentage of White evangelicals have higher mortality rates and more persons with fair/poor health. The potential influence of antistructural components in evangelical theology on decision making and resource allocation and, ultimately, the length and quality of life of community members presents a point of intervention for religious leaders and policymakers to improve population health.

**Context:**

Structural factors are important determinants of health. Because antistructuralism has been identified as a tenet of White evangelical theology, we explored if there is an association of the percentage of White evangelicals in a US county with two county health outcomes: premature mortality and percentage of fair/poor health.

**Methods:**

Regression analysis was performed with data from 2022 County Health Rankings and the American Value Atlas from the Public Religion Research Institute.

**Findings:**

Every percent of evangelicals in a county is associated with 4.01 more premature deaths per 100,000 population and 0.13% fair/poor health. After controlling for income, education, political ideology, and county school funding adequacy (a proxy for antistructuralism), the associations remain positive and significant.

**Conclusions:**

We hope these findings could inform dialogue and critical analysis among individuals of evangelical faith, particularly fundamental and Pentecostal subsets, regarding a belief system that is inclusive of individual dimensions and health‐promoting structural policies like school funding, Medicaid expansion, and antipoverty programs. These findings also demonstrate the importance of considering cultural factors like religion and political ideology in population health outcomes research.

Population health scholars have often suggested expanding the list of health determinants to include cultural, political, or religious factors, which traditional metrics do not often cover. In his book called *White Too Long: The Legacy of White Supremacy in American Christianity*,^1^ Robert Jones, a former Mississippi evangelical pastor with a PhD in theology from Emory University, discusses the remarkable power of White evangelical Christian culture in the theology that undergirds it. He discusses the White evangelical “cultural tool kit,” consisting of strong views that restricts evangelicals’ moral vision to the personal and interpersonal realms while not recognizing or valuing institutional or structural issues. He points out that there are three main tools that are connected by theology: free will individualism, relationalism, and antistructuralism. He observes the following:

…free will individualism means that for white evangelicals, individuals exist independent of structures and institutions, have free will, and are individually accountable for their own actions. Relationalism means that white evangelicals tend to see the root of all problems in poor relationships between individuals rather than in unfair laws or institutional behavior. Finally, anti‐structuralism denotes the deep suspicion with which white evangelicals view institutional explanations for social problems, principally because they believe invoking social structures shifts blame from where it belongs with sinful individuals.

Similarly, Putnam and Campbell observe that compared with other affiliations, evangelicals “are less likely to perceive systemic causes of racism, and thus less likely to endorse systemic efforts to ameliorate its consequences.”^2^

Previous work has also explored this relationship between evangelical beliefs and health outcomes. Blanchard and colleagues note that “the discipline of sociology finds its roots in Durkheim's canonical study on the link between religious structure and the rates of suicide mortality.”^3^ Using 2000 data on county congregations and 1998–2002 NCHS mortality data,^4^ they found a strong significant association between the presence of Conservative Protestant congregations and a higher mortality rate (65.14 per 100,000). They also show that this effect is limited to Pentecostal and fundamentalist conservative congregations as distinct from those they identify as evangelical. They hypothesize that the difference between “worldly and otherworldly mortality theodicies exert strong cultural willingness to invest in a public health infrastructure.”

Additionally, Bartkowski and colleagues examined the impact of congregational type in county infant mortality. Using 2000 Kids Count data with the 2000 Glenmary Census of Churches/Congregations, a high prevalence of Catholic and most types of Conservative Protestant churches were associated with lower rates of infant mortality when compared with counties that feature fewer Catholic and Conservative Protestant congregations. However, communities with a large proportion of Pentecostal churches exhibited significantly higher infant mortality rates.^5^

Given the importance of social structures in population health and health equity,^6^ we explore further here the relationship among religious affiliation, two health outcomes, and other demographic characteristics given a unique and never previously used data set from the Public Religion Research Institute (PRRI). We hypothesize that county‐level evangelical affiliation is associated with higher mortality and morbidity because of antistructuralism proxied here by one specific manifestation: inadequate school funding. We also explore confounding factors such as socioeconomic status and political affiliation. Although we hypothesize the direction of the relationship to be from evangelical affiliation to poor health through antistructuralism, the opposite direction is of course possible to some degree. One study supports the possibility that persons in poor health could be attracted to evangelical theology in the hope of divine intervention.^7^ In addition, Jones (written communication, 2022) notes the following:

…evangelicals are still less likely than other whites to have college degrees, which is also associated with poorer health outcomes. And lower school funding outcomes are likely linked to opposition to Brown v Board of Education. White evangelicals largely pulled their kids out of school en masse in protest of desegregation and established their own (all‐white) private Christian academies, thereby removing their incentive to support public education.

We are hopeful that these findings will be useful to evangelical religious leaders and public policymakers who are interested in improving the health of all communities and congregations.

## Data/Methods

**Figure 1:**
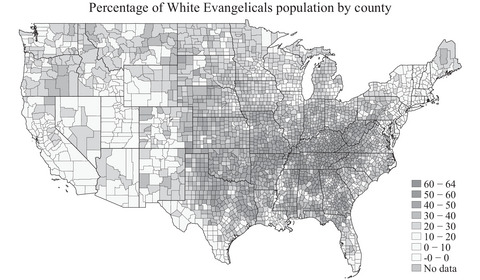
Percentage of White Evangelicals by US County 2020

Our primary exposure of interest is White evangelical: the estimated percentage of county population that self‐identifies as White evangelical Protestant. For data on county religious affiliation, we used the 2020 Census of American Religion by the PRRI.^8^ The designation of evangelical was based on self‐identification—“do you consider yourself an evangelical or born‐again Christian?”—without decomposition including being Pentecostal or fundamentalist, as done by Blanchard and colleagues.^4^ Jones indicates, however, that “generally, the overwhelming majority of Pentecostal/fundamentalists consider themselves to be born again Christians.”^7^ PRRI worked with the National Opinion Research Center to take their American Values Atlas 2013–2019 survey data and generate county‐level estimates for each religious affiliation^8^ using a standard small area estimation approach referred to as “Empirical Best Linear Unbiased Prediction” (Appendix 3). Figure 1 displays the geographic distribution of this religious affiliation by county, with the largest percentages in the Southeast region.

Our primary outcome of interest is premature age‐adjusted mortality: the number of deaths among residents younger than 75 years of age in a county per 100,000 population (age adjusted). Additionally, we test a secondary outcome of interest, poor or fair health: the percentage of adults in a county reporting fair or poor health (age adjusted).

We include three confounding variables: income, education, and political ideology. To measure income, we use median household income: the income in which half of households in a county earn more and half of households in a county earn less ($1,000). To quantify education, we use high school completion: the percentage of adults 25 years old and older in a county with a high school diploma or equivalent. To assess political ideology, we include percentage conservative: the percentage of each county's population that voted for Donald Trump in the 2016 presidential election.

Finally, we assess one potential mechanism: school funding adequacy. School funding adequacy is the average gap in $1,000 between actual and required spending per pupil among public school districts within a county.^9^ Required spending is an estimate of dollars needed to achieve US average test scores in each school district. Because we know that many school funding decisions are made at the state level, we include a random intercept for state in all models that include school funding adequacy.

Data on health outcomes and covariates including school funding adequacy were taken from the County Health Rankings 2022 data set.^10^ County political ideology was measured as percentage voting for Trump 2016 from the Massachusetts Institute of Technology Election Data and Science Lab data set.^11^ Statistical analyses and mapping completed using Stata and R. Summary statistics are presented in Appendix 1.

## Results/Findings

Bivariate regressions reveal that a 1% increment of percentage evangelical in a county is associated with 4.01 more premature deaths per 100,000 population than the overall population. The opposite relationship is seen for the percentage White other Protestant and White Catholics, with 6.3 and 7.4 fewer premature deaths, respectively. For bivariate comparison, every $1,000 of median household income is associated with 5.9 fewer premature deaths and 1% voting for Trump with 1.7 fewer premature deaths. For additional comparison, a 1% increment in smoking rate is associated with 21.7 more premature deaths per 100,000. Similar bivariate relationships are seen for county percentage fair/poor health.

Because of the antistructural components of evangelical theology, we hypothesize that higher percentages of persons with White evangelical affiliation would be associated with worse health outcomes even after adjusting for known confounders. Because education and income are strongly related to theological beliefs as well as health, we include the percentage of the population 25 years old and older with a high school diploma or equivalent and median household income as confounding variables in our baseline model. Additionally, because we aim to isolate potential effects of religious beliefs from potential effects of political ideology, we include the percentage Republican as a confounder in our model. There are of course many other variables that could confound the relationship between religious affiliation and county‐level health. We have chosen median household income, percentage completed high school, and percentage Republican as three that we view as potentially most important. This baseline model (model 1) is shown in Table 1.

**Table 1:** Regression Analysis of Premature Mortality

	Model 1: Number of Deaths Among Residents Age <75 Years per 100,000 (Age Adjusted)			Model 2: Number of Deaths Among Residents Age <75 Years per 100,000 (Age Adjusted)			Model 3: Number of Deaths Among Residents Age <75 Years per 100,000 (Age Adjusted)			Model 4: Number of Deaths Among Residents Age <75 Years per 100,000 (Age Adjusted)		
Predictors	Estimates	CI	*p*	Estimates	CI	*p*	Estimates	CI	*p*	Estimates	CI	*p*
(Intercept)	1,227.32	1,180.11‐1,274.52	<0.001	1,011.65	960.50‐1,062.80	<0.001	883.77	828.55‐938.99	<0.001	826.88	768.52‐885.25	<0.001
Median household income ($1,000)	−4.81	−5.06 to −4.56	<0.001	−4.18	−4.42 to −3.93	<0.001	−3.67	−3.93 to −3.41	<0.001	−3.72	−4.00 to −3.45	<0.001
Percentage of the population who voted for the Republican nominee in the 2016 presidential election	−1.27	−1.68 to −0.86	<0.001	−4.19	−4.70 to −3.68	<0.001	−3.51	−4.03 to −3.00	<0.001	−2.60	−3.17 to −2.02	<0.001
Percentage of adults age ≥25 years with a high school diploma	−5.54	−6.17 to −4.90	<0.001	−3.58	−4.22 to −2.93	<0.001	−2.60	−3.26 to −1.94	<0.001	−1.82	−2.51 to −1.14	<0.001
Percentage White evangelical				2.92	2.59‐3.25	<0.001	2.70	2.38‐3.03	<0.001	1.74	1.30‐2.18	<0.001
School funding adequacy ($1,000 per pupil)							−4.10	−4.84 to −3.36	<0.001	−5.08	−6.08 to −4.08	<0.001
Random effects												
σ^2^										5,430.72		
τ_00_										1,218.11_state_		
ICC										0.18		
*N*										50_state_		
Observations	3,004			3,004			3,004			3,004		
R^2^/R^2^ adjusted	0.539/0.538			0.581/0.581			0.597/0.596			0.550/0.632		

CI, confidence interval; ICC, intraclass correlation coefficient.

When we add percentage county population that self‐identifies as White evangelical Protestant (model 2), we see that the coefficient for percentage White evangelical remains statistically significantly different from 0% even after accounting for county‐level median household income, percentage completed high school, and percentage voting Republican, suggesting that county‐level White evangelical affiliation is meaningful when trying to explain county‐level differences in health. After adjusting for income, education, and political ideology, an increase of 1% White evangelical in a county is associated with 2.92 additional premature deaths per 100,000 population.

We hypothesize that the relationship between evangelical beliefs and county‐level premature mortality might be explained by evangelical antistructural theology. A measurable proxy for antistructuralism for which data are available is school funding adequacy: the average gap in dollars between actual and required spending per pupil among public school districts within a county. Thus, we add school funding adequacy (model 3). Because we know that many school funding decisions are made at the state/national level rather than the county level,^12^ model 4 includes a random intercept for the state to account for such differences. After accounting for school funding adequacy and state‐level differences in addition to confounding caused by income, education, and political ideology, we find that a 1% increase in county‐level percentage White evangelical is associated with a 1.74 increase in premature deaths per 100,000 population.

Table 2 shows the results for a similar regression for the outcome of self‐reported fair or poor health, which ranges from 8.9% to 44.8% across all counties.

**Table 2:** Regression Results for the Percentage of Fair/Poor Health

	Model 1: Percentage of Adults Reporting Fair or Poor Health (Age Adjusted)			Model 2: Percentage of Adults Reporting Fair or Poor Health (Age Adjusted)			Model 3: Percentage of Adults Reporting Fair or Poor Health (Age Adjusted)			Model 4: Percentage of Adults Reporting Fair or Poor Health (Age Adjusted)		
Predictors	Estimates	CI	*p*	Estimates	CI	*p*	Estimates	CI	*p*	Estimates	CI	*p*
(Intercept)	77.75	76.63‐78.86	<0.001	73.07	71.86‐74.28	<0.001	68.73	67.45‐70.01	<0.001	63.73	62.57‐64.89	<0.001
Median household income ($1,000)	−0.12	−0.13 to −0.12	<0.001	−0.11	−0.11 to −0.10	<0.001	−0.09	−0.10 to −0.09	<0.001	−0.10	−0.11 to −0.10	<0.001
Percentage of the population who voted for the Republican nominee in the 2016 presidential election	−0.08	−0.09 to −0.07	<0.001	−0.14	−0.16 to −0.13	<0.001	−0.12	−0.13 to −0.11	<0.001	−0.06	−0.07 to −0.05	<0.001
Percentage of adults age ≥25 years with a high school diploma	−0.55	−0.56 to −0.53	<0.001	−0.50	−0.52 to −0.49	<0.001	−0.47	−0.49 to −0.45	<0.001	−0.40	−0.42 to −0.39	<0.001
Percentage White evangelical				0.06	0.06‐0.07	<0.001	0.06	0.05‐0.06	<0.001	−0.02	−0.03 to −0.01	<0.001
School funding adequacy ($1,000 per pupil)							−0.14	−0.16 to −0.12	<0.001	−0.15	−0.17 to −0.13	<0.001
Random effects												
σ^2^										1.89		
τ_00_										2.17_state_		
ICC										0.53		
*N*										50_state_		
Observations	3,004			3,004			3,004			3,004		
R^2^/R^2^ adjusted	0.837/0.837			0.849/0.849			0.861/0.861			0.790/0.902		

CI, confidence interval; ICC, intraclass correlation coefficient.

Similar relationships across the variables are seen as for premature mortality. After adjusting for income, education, and political ideology (model 1), a 1% increase in percentage White evangelical (model 2) is associated with an increase of 0.06% of the population reporting fair/poor health. Controlling for school funding adequacy and state‐level differences (model 4), a 1% increase in White evangelical population is associated with an average decrease of 0.02% of the population reporting fair/poor health. A $1,000 increase in school funding adequacy is associated with an average of 0.15% decrease in the percentage of the population reporting fair/poor health.

## Limitations

Because we use ecological county‐level data, interpretations of specific associations between the variables and mortality changes should not be made at the level of the individual.^13^ However, ecologic analyses are valuable to determine the role of upstream factors, such as social and environmental policies, on health and are often the method of choice when public health action is being considered.^13^, ^14^ All of our results are associations that can suggest, but could not of course prove, causal relationships.

There is some survey and modeling error associated with the estimates of religious affiliation. The data set was created using 5 years of survey data, totaling more than 450,000 cases, plus additional data from the American Communities Survey in a small area estimation model to arrive at the county‐level estimates for each religious group. Larger population counties, which have more survey data, will have more precise estimates than smaller counties with less survey data. Details on the surveys and modeling processes are in Appendices 2 and 3.

The survey data are limited to a single self‐report category of evangelicals without decomposition for fundamentalists/Pentecostals, as done for congregations by Blanchard and colleagues.^4^ Because lower mortality in their study was associated only with those two subgroups, that could likely be the case with our results. Although decomposition, if possible, might therefore change our results, the impact of antistructuralism could then increase the impact from fundamentalist/Pentecostal groups. Additionally, evangelicals who are Black are included in the Black Protestant category, and evangelicals who are Hispanic are included in the Hispanic Protestant category. This is because of the small sample sizes in the underlying surveys, and it means that the results here are speaking only to White evangelicals and not capturing possible effects among other race evangelicals. Further work is needed to further understand these issues.

Because the regressions are linear, it is not clear that each percentage increase in evangelicals has the same association at different percentages; the association with health outcomes at each percentage of evangelicals could be nonlinear (i.e., not the same at 40% vs. 70%).

We acknowledge that school funding adequacy is only one potential proxy for evangelical antistructuralism.

Our state effect results show that the effect of local school funding adequacy is reduced but still significant. Further research is needed to identify other antistructural policies that could also be relevant.

## Discussion/Conclusions

The answer to the question posed in the title is yes; there is a correlation between county White evangelical affiliation and poor health measured by premature mortality and fair/poor health. The relationships described hold after controlling for other potential confounders such as political ideology and socioeconomic status, and opposite bivariate relationships are seen for two large alternate affiliations: nonevangelical Protestants and Catholics.

What might the mechanism of this association be? Our suggestion that the antistructural element of evangelical theology may be at least partially responsible is supported when our suggested measure of county school funding adequacy is added to the regression equation. School funding is substantially a local and state institutional decision that certainly could be impacted by evangelical legislators and county board members. A similar but much smaller relationship was seen for county access to exercise opportunities (not shown) that could also reflect local antistructural policy perspective.

How important is this relationship in explaining county health? Bivariate relationships with county premature mortality show that for every increment of 1% of evangelicals in a county, there are about four more premature deaths. In comparison, 1% county smoking rate is associated with 22 more premature deaths. Applying this to an individual county, a county with the average smoking rate of 12% would result in 264/100,000 more premature deaths; if our results were causal, a county with 50% evangelicals, enough to impact local institutional policies would result in 200/100,000 more premature deaths.

Religious affiliation and political ideology are commonly thought to be closely related. It has often been thought that early family religion affiliation produces future political ideology, but recently, Margolis has proposed a life cycle theory in which religious identities are in part a response to their political surrounding, especially in adolescence.^15^ In these data, the correlation between percentage conservative voters and percentage evangelicals is 0.75, whereas with other Protestant, it is 0.43, and with Catholic, it is −0.17. The direction of these correlations is not clear. Surprisingly, the percentage conservative is associated with fewer premature deaths and fair/poor health, whereas percentage evangelical is associated with more deaths and fair/poor health. Further research is needed to explain these relationships.

We hope that this study adds to the quest for finding the full range of determinants of health, including cultural factors such as religion, political ideology, and power seldom currently reported, called the Fantasy Equation in 1995 by Stoddart. He then stated that “at present we but vaguely understand the relative magnitude of the coefficients on the independent variables that would inform specific policies rather than broad directions, even if we are beginning to see the variables themselves more clearly.”^16^ We encourage additional work with other cultural variables and methods to add our understanding of what makes us healthier.

What is the policy relevance of these results? We do not know how strongly an antistructural component of evangelical theology is held by members and pastors and the organized affiliation. This must vary considerably across individuals and congregations, with some strongly adhering and others being only sympathetic, and others not at all. It may differ across generations and is likely changing over time as the number of evangelicals declines.^17^

Previous research on congregations instead of individuals found that similar results were only found for two subsets of evangelicals.^4^ Bartkowski and colleagues have noted that during that during the COVID‐19 pandemic, some leading evangelicals directly challenged religious conservative misinformation efforts undertaken by prominent conservative Protestants. They suggest that it is possible that public health information might be better received in evangelical (world‐engaging) congregations than in fundamentalist (world‐condemning) and Pentecostal (faith‐healing) congregations. Additionally, some high‐profile evangelicals are clearly receptive to the lessons learned from pandemic science. So, perhaps the building of selective alliances and strategic dialogues with the evangelical wing of conservative Protestantism could provide a path forward for public health advocacy.^18^ Further research should explore these dimensions of such relationships.

We would hope that these findings, presented in ways such as the previous example comparing the similarity between the health effects of smoking and antistructural beliefs might bring the importance of structural policies like school funding, Medicaid expansion and antipoverty program into mainstream discussion among policymakers, religious leaders, advocates, and citizens.

## Conflict of Interest Disclosures

No disclosures were reported.

## Supporting information

Appendix
