# Supplementary material for: Is White Evangelical Antistructural Theology Related to Poor Health Outcomes?
Source: Milbank Q. 2024 Jan 19;102(2):503–16. doi: 10.1111/1468-0009.12688 (PMC11176399; doi:10.1111/1468-0009.12688)
Supplement: Supplementary file 1 — Appendix [file MILQ-102-503-s001.docx]

**Appendix 1**

**Descriptive Statistics for All Dependent and Independent Variables (*N* = 3,115)**

| Variables | N | Mean (SD) | Range (min-max) | Notes |
| --- | --- | --- | --- | --- |
| White Evangelical | 3,115 | 30.32 (12.86) | 0 – 64 | Percent of the county population who are White evangelicals. |
| School Funding Adequacy | 3,037 | -.20 (5.13) | -22.20 – 26.23 | In $1000 dollars, range from deficit (negative values) to adequacy (positive values). |
| Median Household Income | 3,115 | 57.35 (14.53) | 22.90 – 160.31 | Median value of the county, in $1000 dollars. |
| Conservative | 3,115 | 63.24 (15.70) | 4.09 – 94.58 | Percent of the county population who voted for Trump in the 2016 election |
| Premature Mortality | 3,060 | 431.47 (127.06) | 133.03 – 1304.32 | Number of premature deaths per 100,000 age adjusted population. |
| Fair & Poor Health | 3,115 | 20.62 (5.01) | 8.9 – 44.8 | Percent of the county population who reported fair/poor health. |

**Appendix 2**

**American Values Atlas Survey Methodology**

The 2013-2019 American Values Atlas (AVA) is a project of PRRI. Results for all demographic, religious affiliation, and political affiliation questions were based on 53,474 bilingual telephone interviews (including 33,708 cell phone interviews) conducted between January 2, 2019, and December 30, 2019, by professional interviewers under the direction of SSRS. The complete 2013-2019 dataset contains 453,822 interviews. The survey was made possible by generous support from the **Arcus Foundation**, **the E. Rhodes & Leona B. Carpenter Foundation, the Evelyn and Walter Haas**, **Jr. Fund**, **the Gill Foundation**, and **Unitarian Universalist Veatch Program at Shelter Rock**.

Each year, at least 1,000 interviews were completed each week, with percentages of cell phone interviews increasing over the years to 70% in 2019. Each week, interviewing occurred over a five- or six-day period, starting Tuesdays or Wednesdays and going through Sunday or Monday. The selection of respondents within households was accomplished by randomly requesting to speak with the youngest adult male or female currently living in the household.

Data collection was based on stratified, single-stage, random-digit-dialing (RDD) of landline telephone households and randomly generated cell phone numbers. The sample was designed to represent the total U.S. adult population from all 50 states, including Hawaii and Alaska, and the District of Columbia. The landline and cell phone samples were provided by Marketing Systems Group.

The weighting was accomplished in two separate stages. The first stage of weighting corrects for different probabilities of selection associated with the number of adults in each household and each respondent’s telephone usage patterns. In the second stage, sample demographics were balanced to match target population parameters for gender, age, education, race and Hispanic ethnicity, region (U.S. Census definitions), population density, and telephone usage. The population density parameter was derived from 2010 Census data. The telephone usage parameter came from an analysis of the National Health Interview Survey. All other weighting parameters were derived from an analysis of the U.S. Census Bureau’s Current Population Survey.

The sample weighting was accomplished using iterative proportional fitting (IFP), a process that simultaneously balances the distributions of all variables. Weights are trimmed so that they do not exceed 4.0 or fall below 0.25 to prevent individual interviews from having too much influence on the final results. The use of these weights in statistical analysis ensures that the demographic characteristics of the sample closely approximate the demographic characteristics of the target populations.

**Appendix 3**

**County-Level Model Methodology**

PRRI worked with NORC to take the American Values Atlas survey data and generate county-level estimates for each religious affiliation using a technique called small area estimation modeling. NORC has developed and implemented small area models on a number of key projects for government and social science surveys.

The small area modeling approach for this project entailed modeling weighted survey estimates of each religious denomination by county and year. Since the distribution of religious denominations are correlated across time, the small area model captures county-level correlations across time via a time-series component to improve estimates for the most current year. Furthermore, the model incorporates external auxiliary data (that is predictive of the distribution of religious denominations) at the county-level from the American Community Survey. The best set of predictors for each religious denomination was selected using a variable selection approach known as LASSO. Model-based estimates for each religious denomination and county were generated using a standard small area estimation approach referred to as “Empirical Best Linear Unbiased Prediction”. Finally, model-based estimates for each religious denomination were generated for all counties in the U.S., not limited to counties with survey data.
